# The complete mitogenome and phylogenetic analysis of *Acrossocheilus beijiangensis* (osteichthyes: Cyprinidae)

**DOI:** 10.1080/23802359.2017.1413314

**Published:** 2017-12-16

**Authors:** Xiao-Xiang Liu, Xue-Lin Song, Hong-Long Li, Le-Yang Yuan

**Affiliations:** a Institute of Hydrobiology, Chinese Academy of Sciences, Wuhan, Hubei, China;; b Faculty of Basic Medicine, Hangzhou Medical College, Hangzhou, Zhejiang, China;; c University of Chinese Academy of Sciences, Beijing, China;; d Dalian Natural History Museum, Dalian, Liaoning, China;; e Zhejiang Museum of Natural History, Hangzhou, Zhejiang, China;; f Biodiversity Research Center of Zhejiang Province, Hangzhou, Zhejiang, China

**Keywords:** Barred species, mitogenome, *Acorssocheilus beijiangensis*, next-generation sequencing

## Abstract

*Acorssocheilus beijiangensis* is an endemic south China stream-dwelling cyprinid species. Its complete mitochondrial genome is 16,596 bp in length, consisting of 13 protein-coding genes, 22 tRNA genes (ranging from 67 bp in *tRNA^Cys^* to 76 bp in *tRNA^Leu^* and *tRNA^Lys^*), two rRNA genes (959 bp in 12S rRNA and 1683 bp in 16S rRNA), and one control region (937 bp). Its overall base composition is A: 31.1%, C: 27.9%, G: 16.2%, and T: 124.8%. The complete mitogenome of the Chinese barred species of Cyprinidae could provide a basic data for further phylogenetics analysis.

*Acrossocheilus beijiangensis* is a barred cyprinid species from the Pearl River (Zhu-Jiang) of Guizhou, Guangxi and Guangdong provinces in China (Wu et al. 1977; Shan et al. 2000). This species differs from the congenus with a combination of characters: five vertical bars on the flanks 2–4 scales wide extending slightly beyond the lateral line; the second bar positioned posterior to the base of the last simple dorsal-fin ray; no longitudinal stripe along the lateral line; two thick lateral lobes of the lower lip; last unbranched dorsal-fin ray stout with a serrated posterior edge; membranes between the dorsal-fin rays oblong with black blotches. However, ontogenetic changes and/or sexual dimorphism in body coloration and mouthpart structures usually confuse researchers when delineating these barred species. Thus, combination of mitochondrial DNA data and morphological traits will help for the species identification of taxonomic validity and phylogenetic position of the species.

The sample caught from the Congjiang county (Duliu-Jiang, flowing to the Xi-Jiang River) of Guizhou Province in China was deposited in the collection of the Zhejiang Museum of Natural History under the accession number ZMNH 2011081501. The complete mitogenome of *Acorssocheilus beijiangensis* has been obtained from high-throughput sequencing on whole-genomic DNA with HiSeq 2000 platform (Illumina, San Diego, CA). We used next-generation sequencing to perform low-coverage whole-genome sequencing according to previous protocol (Shen et al. 2016). The complete mitogenome of *A. beijiangensis* is 16,596 bp in length (GenBank KY131976), includes 13 protein-coding genes, 22 tRNA genes (ranging from 67 bp in *tRNA^Cys^* to 76 bp in *tRNA^Leu^* and *tRNA^Lys^*), two rRNA genes (959 bp in 12S rRNA and 1683 bp in 16S rRNA) and one D-loop control region (937 bp). Its overall base composition is A: 31.1%, C: 27.9%, G: 16.2%, and T: 24.8% which shows AT bias, with the AT content of 55.9%. The complete mitogenome of *A. beijiangensis* showing 97% identities to *A. stenotaeniatus* (GenBank KJ909660) after BLAST search against NCBI nr/nt database.

To validate the phylogenetic position of *A. beijiangensis*, we used MEGA6 software (Tamura et al. 2013) to construct a maximum-likelihood tree (with 100 bootstrap replicates and Kimura 2-parameter model) containing complete mitogenomes of 12 species derived from *Acrossocheilus* genus. *Onychostoma gerlachi* (Cheng et al. 2016) derived from *Onychostoma* genus was used as outgroup for tree rooting (Figure 1). The resultant phylogeny shows that the *A. beijiangensis* is closely related to *A. stenotaeniatus* with high bootstrap value supported. The Kimura-two-parameter distance was 0.029 between *A. beijiangensis* and *A. stenotaeniatus* and was 0.074 between *A. beijiangensis* and *A. wuyiensis*. The complete mitogenome of *A. beijiangensis* which deduced in this study provides a basic data for further phylogenetic and conversational analysis of the Chinese barred species in Cyprinidae.

**Figure 1.**
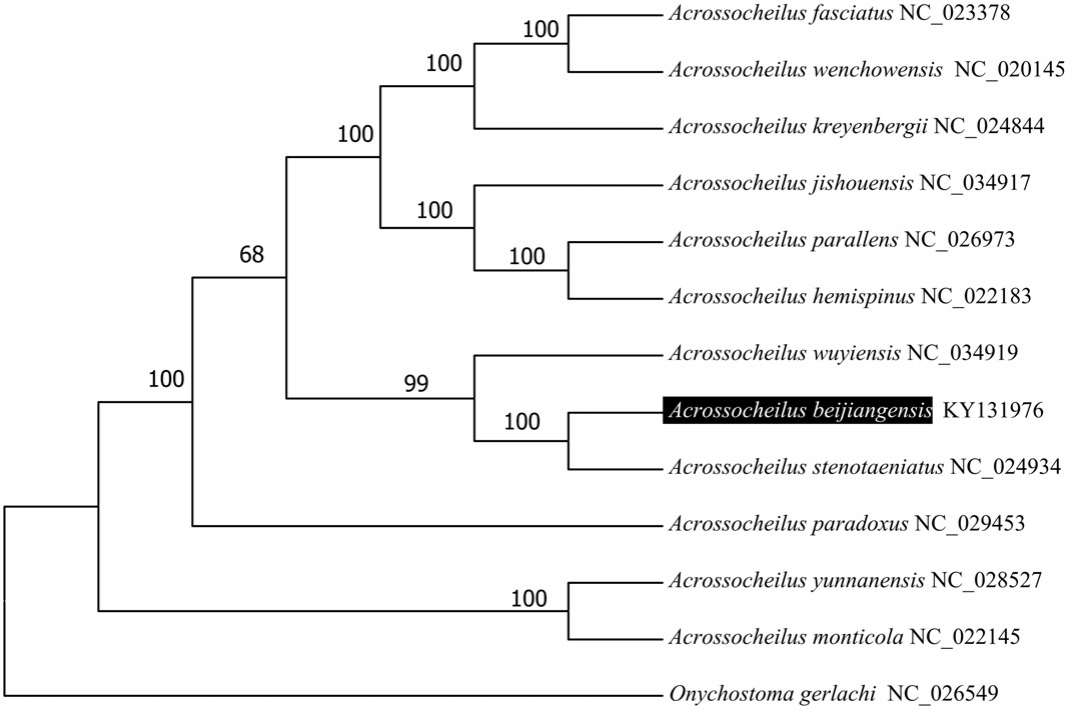
Molecular phylogeny of *Acrossocheilus beijiangensis* and related species based on complete mitogenome. The complete mitogenomes is downloaded from GenBank and the phylogenic tree is constructed by maximum-likelihood method with 500 bootstrap replicates. The gene's accession number for tree construction is listed behind the species name.
